# Live or Heat-Killed *Lactobacillus rhamnosus* Aerosolization Decreases Adenomatous Lung Cancer Development in a Mouse Carcinogen-Induced Tumor Model

**DOI:** 10.3390/ijms232112748

**Published:** 2022-10-22

**Authors:** Valentino Le Noci, Giancarla Bernardo, Giacomo Manenti, Gabriele Infante, Dariush Khaleghi Hashemian, Lucia Minoli, Simone Canesi, Francesca Bianchi, Tiziana Triulzi, Stefania Arioli, Loris De Cecco, Simone Guglielmetti, Federico Ambrogi, Camilla Recordati, Nicoletta Gagliano, Elda Tagliabue, Michele Sommariva, Lucia Sfondrini

**Affiliations:** 1 Dipartimento di Scienze Biomediche per la Salute, Università degli Studi di Milano, 20133 Milan, Italy; 2 Animal Health and Welfare Unit, Department of Applied Research and Technical Development, Fondazione IRCCS Istituto Nazionale Tumori, 20133 Milan, Italy; 3 Laboratory of Medical Statistics and Biometry “Giulio A. Maccacaro”, Department of Clinical Sciences and Community Health, Università degli Studi di Milano, 20122 Milan, Italy; 4 Unit of Clinical Epidemiology and Trial Organization, Department of Applied Research and Technological Development, Fondazione IRCCS Istituto Nazionale dei Tumori, 20133 Milan, Italy; 5 Dipartimento di Scienze Veterinarie, Università degli Studi di Torino, 10095 Turin, Italy; 6 Mouse and Animal Pathology Laboratory (MAPLab), Fondazione Unimi, 20139 Milan, Italy; 7 Dipartimento di Medicina Veterinaria e Scienze Animali, Università degli Studi di Milano, 26900 Lodi, Italy; 8 U.O. Laboratorio di Morfologia Umana Applicata, IRCCS Policlinico San Donato, 20097 San Donato Milanese, Italy; 9 Molecular Targeting Unit, Department of Research, Fondazione IRCCS Istituto Nazionale dei Tumori, 20133 Milan, Italy; 10 Dipartimento di Scienze per gli Alimenti, la Nutrizione e l’Ambiente (DeFENS), Università degli Studi di Milano, 20133 Milan, Italy; 11 Molecular Mechanisms Unit, Department of Research, Fondazione IRCCS Istituto Nazionale dei Tumori, 20133 Milan, Italy; 12 Scientific Directorate, IRCCS Policlinico San Donato, 20097 San Donato Milanese, Italy

**Keywords:** carcinogenesis, lung cancer, lung microbiota, *Lactobacillus rhamnosus*, mouse models, tumor prevention, aerosol, urethane, J chain, IgA

## Abstract

An immunosuppressive microenvironment in lung concurs to pre-malignant lesions progression to cancer. Here, we explore if perturbing lung microbiota, which contribute to immunosuppression, by antibiotics or probiotic aerosol interferes with lung cancer development in a mouse carcinogen-induced tumor model. Urethane-injected mice were vancomycin/neomycin (V/N)-aerosolized or live or dead *L. rhamnosus GG* (*L.RGG*)-aerosolized, and tumor development was evaluated. Transcriptional profiling of lungs and IHC were performed. Tumor nodules number, diameter and area were reduced by live or heat-killed *L.RGG*, while only a decrease in nodule diameter was observed in V/N-treated lungs. Both *L.RGG* and V/N reduced Tregs in the lung. In *L.RGG*-treated groups, the gene encoding the joining chain (J chain) of immunoglobulins was increased, and higher J chain protein and IgA levels were observed. An increased infiltration of B, NK and myeloid-derived cells was predicted by TIMER 2.0. The Kaplan–Meier plotter revealed an association between high levels of J chain mRNA and good prognosis in lung adenocarcinoma patients that correlated with increased B and CD4 T cells and reduced Tregs and M2 macrophages. This study highlights *L.RGG* aerosol efficacy in impairing lung cancer growth by promoting local immunity and points to this non-invasive strategy to treat individuals at risk of lung cancer.

## 1. Introduction

Lung cancer is one of the leading causes of cancer death around the world [1]. Despite the introduction of novel, more effective chemo/immunotherapeutic options, it remains an incurable disease, with an overall 5-year survival rate of 15% for men and 21% for women [2]. In the majority of patients, due to the lack of early-stage clinical evidence, diagnosis occurs in an advanced stage of disease. The introduction of screening with low-dose computed tomography (LDCT) has proven to be effective in detecting early-stage disease in high-risk populations, such as smokers and former smokers [3,4], allowing, when possible, surgical treatment. However, even in resected patients, recurrence rates remain very high: from 30% for stage I to 80% for stage III disease. Nodules detected by LDCT that are considered at low risk of being cancer may undergo repeated CT scans within a designated time interval for monitoring. If nodules remain stable in size over a 2-year period, they are generally considered to be benign. Thus, novel strategies able to reduce the risk of recurrence in early-stage resected patients or the progression of low-risk nodules during the monitoring period might be highly beneficial.

Lung cancer progresses through a series of pre-malignant histologic changes, defined by genetic and epigenetic alterations in pulmonary epithelial cells, before the development of invasive disease. Approximately 85% of lung cancer cases are related to tobacco smoke, which contains both direct carcinogens and agents that promote the growth of nascent tumors [5,6], and to environmental carcinogens, such as asbestos, arsenic, radon and air pollution. These compounds may act synergistically as lung carcinogens, tumor promoters and cocarcinogens [7,8,9,10] by inducing the production of reactive oxygen species (ROS) and DNA/chromosomal damage and by affecting proteins involved in cell cycle progression and regulation. Although genetic mutations represent a sort of prerequisite for the malignant transformation, their presence is not sufficient for cancer development, and the establishment of an immunosuppressive microenvironment has been considered a necessary factor concurring to the activity of carcinogen and progression from pre-malignant lesions to cancer [11,12]. Indeed, it has been proven that most of the carcinogens trigger pulmonary chronic inflammation, which in turn triggers immune-suppressive mechanisms such as the accumulation of myeloid-derived suppressor cells (MDSC) and regulatory T cell (Tregs), which are able to inhibit the antitumor response and promote tumorigenesis [13,14,15,16]. Rosin et al. demonstrated that the development of the lung nodules in mice after urethane injection, a chemical compound which recapitulates the effect of carcinogens in experimental models, was associated with an increase in the percentage of MDSC and Tregs [17]. Zaynegetdinov et al. found that the absence of alveolar macrophages significantly reduced lung urethane-induced carcinogenesis both at early and late stages of the tumor progression [18]. Additionally, in vivo studies in mice showed that cigarette smoke combined with urethane leads to tumoral lesion development due to the accumulation of MDSCs with a potent immunosuppressive activity [19] or of pulmonary pro-tumor macrophages in lungs [20].

The presence of commensal bacteria in lungs has recently been proposed to play a role in the recruitment and activation of tolerogenic antigen-presenting cells (APCs) and immunosuppressive Treg [21,22,23]. We demonstrated that aerosolization of vancomycin/neomycin (vanco/neo) drastically reduced bacteria of the genus *Streptococcus*, which are common Firmicutes commensal of the respiratory tract. This change was associated with decreased T regs infiltration in the lungs that in turn favored the activation of antitumor effector cells able to prevent experimental lung metastases implantation induced by B16 melanoma cells i.v. injection [24]. We also demonstrated that the immunosuppressive effects of the resident microflora in the lung can be overcome by the aerosolization of *Lactobacillus rhamnosus GG (L.RGG),* a Gram-positive lactic acid bacterium considered a stable commensal of the oral microbiota, by promoting immune effector activations [24]. These data support the use of modulators of lung microbiota by aerosol to influence the local immune microenvironment as a feasible and non-invasive strategy to reduce tumor development in patients at risk of lung cancer development or recurrence.

In the present study, we analyzed the effect of antibiotics or live/heat-killed probiotics aerosolization in a mouse carcinogen-induced tumor model that give rise to bronchioalveolar adenomas that progress to adenocarcinomas and that resemble the subtype of non-small cell lung carcinoma in humans.

## 2. Results

### 2.1. Effect of Aerosolized Vancomycin/Neomycin and Lactobacillus rhamnosus GG on Primary Adenomatous Lung Cancer Development

We assessed whether aerosolization with vanco/neo or with *L.RGG*, live or heat-killed, as a safer approach, could reduce the development of adenocarcinomas induced by urethane carcinogen injection in A/J mice that have a genetic background sensitive to the carcinogen [25].

**Figure 1:**
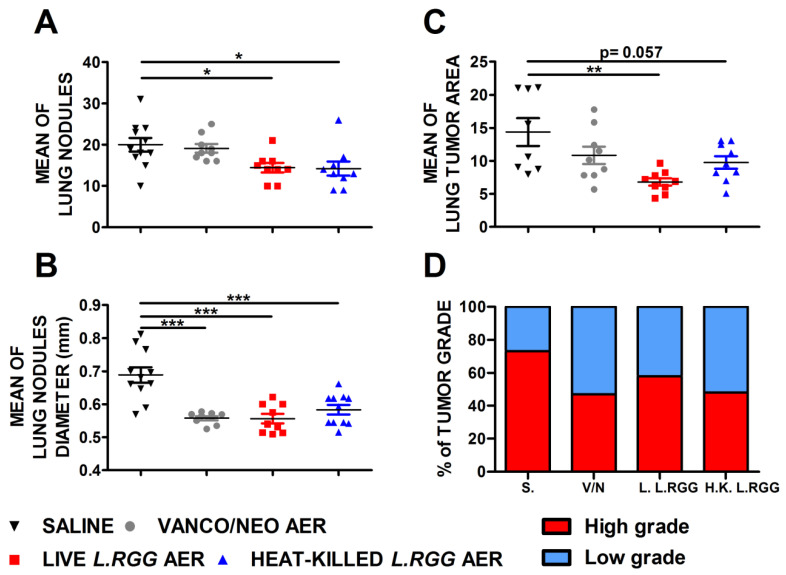
Effect of vancomycin/neomycin or *L.RGG* aerosolization on adenocarcinomas development induced by urethane in highly sensitive A/J mice model. Mean number (**A**), mean diameter (**B**) and mean tumor area (**C**) of tumor nodules in the lungs of A/J mice i.p. injected with urethane and aerosolized with vanco/neo, live *L.RGG* and heat-killed *L.RGG* (9 mice/group) starting two weeks after injection (mean ± SEM). (**D**) Percentages of tumor grade in mice treated with aerosolized antibiotics or probiotic evaluated by H&E staining. High-grade nodules include adenocarcinoma and adenoma with atypia; low-grade nodules include hyperplasia and adenoma. * *p* ≤ 0.05, ** *p* ≤ 0.01 and *** *p* ≤ 0.001 by unpaired Student *t*-test.

The effect on tumor growth was evaluated by measuring both the number and diameter of tumor nodules macroscopically detected on the lung surface and the total tumor area on H&E-stained lung sections. As shown in Figure 1, aerosol treatment with live or heat-killed *L.RGG* induced a statistically significant reduction in the number and diameter of tumor nodules on the lung surface compared to the saline-treated group (Figure 1A,B). Vanco/neo treatment determined a significant shrinkage of the diameters of tumor nodules but did not affect their number. Tumor area was significantly lower in mice administered with live *L.RGG*, and a trend toward a reduction was observed in heat-killed *L.RGG*-treated mice. Vanco/neo aerosolization slightly impacted tumor area (Figure 1C). Interestingly, histological evaluation of tumor samples showed that the percentage of high-grade nodules, including adenocarcinoma and adenoma with atypia, was lowered by all the three treatments compared to controls (Figure 1D and Figure S1).

We previously reported that aerosolized vanco/neo modified lung microbiota composition [24]. Metataxonomic profiling revealed significantly increased α-diversity, estimated in terms of observed OTUs and Chao1 index, also in lungs of live *L.RGG*-treated mice compared to healthy control mice (Figure S2A). No difference in bacterial evenness (Shannon, Simpson and inverse Simpson) was detected (Figure S2A). Control and live *L.RGG*-treated samples did not cluster separately by inter-sample β-diversity analysis (Figure S2B), and 10 taxa were found differentially represented in *L.RGG*-aerosolized versus control mice (Figure S2C).

**Figure 2:**
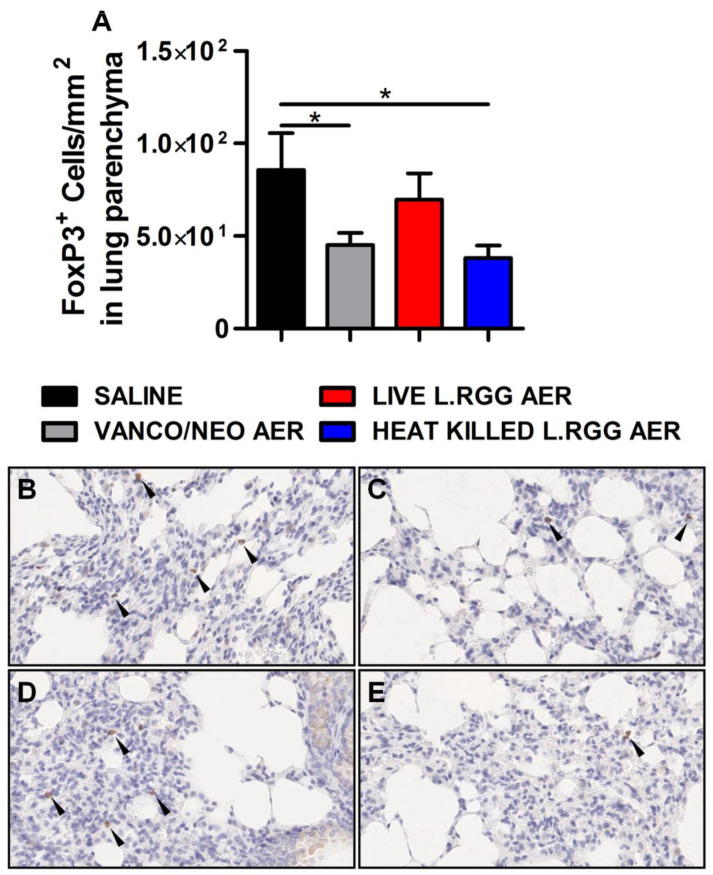
Effect of antibiotics and probiotic aerosol therapy on lung infiltrating T regs. (**A**) FoxP3 protein level in formalin-fixed, paraffin-embedded lung parenchyma specimens evaluated by IHC analysis (4 section/lung, 8–9 mice/group) (mean ± SEM). Data are expressed as positive cells/mm^2^. Representative staining with anti-mouse FoxP3 antibody in (**B**) saline-, (**C**) vanco/neo-, (**D**) live *L.RGG*- and (**E**) heat-killed *L.RGG*-treated lungs are shown. Arrows indicate FoxP3^+^ cells; 40× magnification. * *p* ≤ 0.05 by unpaired Student *t*-test.

Thus, based on our previously published data indicating that the perturbation of lung microbiota following aerosolized antibiotic administration was associated with a reduction in Tregs, FoxP3 expression, a transcriptional factor essential for Tregs functionality [26], was analyzed on formalin-fixed, paraffin-embedded lung sections by IHC. Both vanco/neo and heat-killed *L.RGG* aerosol-treated mice determined a significant reduction of FoxP3^+^ cells in lung parenchyma, while live *L.RGG* only induced a moderate decrease (Figure 2). FoxP3 expression within tumor nodules showed no significant differences among the experimental groups.

A similar experiment performed in BALB/c mice, a strain characterized by less sensitivity to the carcinogen due to the presence of polymorphisms in pulmonary adenoma susceptibility 1 (Pas1) locus [27], revealed a trend of reduction in the development of lung tumors induced by urethane injection, as above. Indeed, tumor nodule numbers was found lower, and diameters of lesions were reduced in vanco/neo and heat-killed *L.RGG*-treated compared to control groups, although not reaching statistical significance (Figure S3A,B). These results could be explained by the fact that tumor incidence at the time of the observation (sixteen weeks) was lower compared to that observed in A/J mice. For instance, a mean number of lesions per mouse less than 2 in 60% of control mice was detected, while in A/J mice, we found an average of 20 lesions per mouse in 100% of mice.

Overall, these data reveal that aerosol treatment with antibiotics or probiotic is able to reduce lung tumor development, and that live or heat-killed *L.RGG* is more effective in reducing both the number and the dimension of tumor lesions. In antibiotic and heat-killed *L.RGG*-treated mice, this effect is associated with a reduced amount of Treg cells infiltrating the lung.

### 2.2. Transcriptional Profiling of Aerosolized Vanco/Neo and L.RGG Adenomatous Cancer Bearing Lungs

To analyze the effects of vanco/neo, live or heat-killed *L.RGG* on the lungs of urethane-injected A/J-treated mice at the molecular level, we performed a comprehensive gene expression profile on RNA extracted from lung specimens. In heat-killed *L.RGG*-treated lungs, eleven genes were found significantly differentially expressed at FDR < 5% compared to saline treatment (six up- and five down-modulated genes) (Table 1). In live *L.RGG*-treated samples, only one gene was found to be up-modulated with FDR < 5%. No genes were significantly different in the vanco/neo- versus saline-treated groups.

Among the differentially expressed genes between heat-killed *L.RGG*- and saline-treated mice, *J chain* gene, a component of secreted immunoglobulins (Ig) [28], showed the highest fold-change (FC: 8.21). Notably, the same gene was the only one significantly modulated in the live *L.RGG*-treated group compared to the control group (FC: 18.49) (Table 2). The other genes reaching the significance threshold in the heat-killed *L.RGG*-treated group were mostly related to genes involved in the signal transmission at the membrane level in different pathways, such as Git1, Vmn1r73 and Scimp (Table 1).

**Figure 3:**
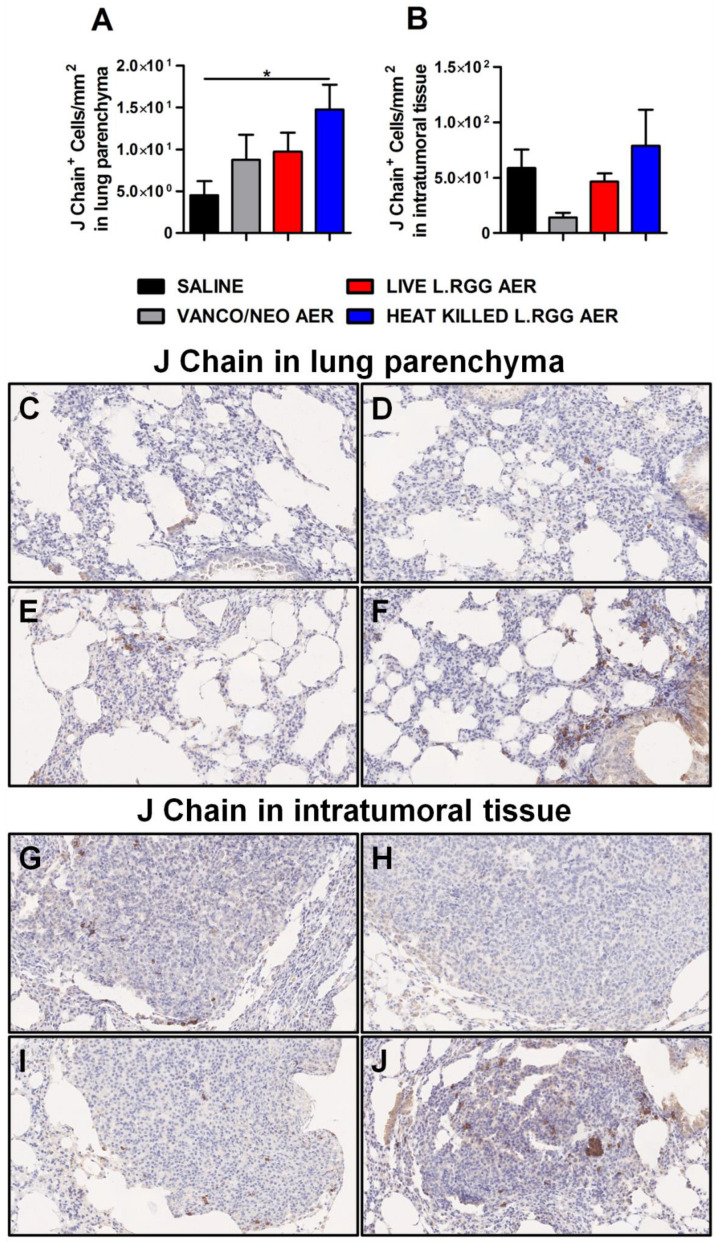
Impact of antibiotics and probiotic aerosolization on J chain protein expression in the lung. Lung parenchyma (**A**) and intratumoral tissue (**B**) J chain protein level in formalin-fixed, paraffin-embedded lung specimens evaluated by IHC. Data are expressed as positive cells/mm^2^ (4 section/lung, 8–9 mice/group) (mean ± SEM). Lung parenchyma and intratumoral tissue representative staining with anti-mouse J chain antibody in saline- (**C**,**G**), vanco/neo- (**D**,**H**), live *L.RGG-* (**E**,**I**) and heat-killed *L.RGG*- (**F**,**J**) treated lungs are shown; 20× magnification. * *p* ≤ 0.05 by unpaired Student *t*-test.

To validate gene profiling data, IHC analysis for J chain expression was performed on formalin-fixed, paraffin-embedded lung specimens obtained from the different experimental groups. According to microarray results, we observed a significant increase in J chain protein level in parenchymal tissue of heat-killed *L.RGG* aerosol-treated mice (Figure 3A,F). Even if not statistically significant, an augmented intensity staining signal of J chain protein was observed in lung samples from vanco/neo and live *L.RGG* aerosol-treated mice compared to control animals (Figure 3A,D,E). J chain did not show expression variations among the different groups when analyzed within tumor nodules (Figure 3B,G–J).

**Figure 4:**
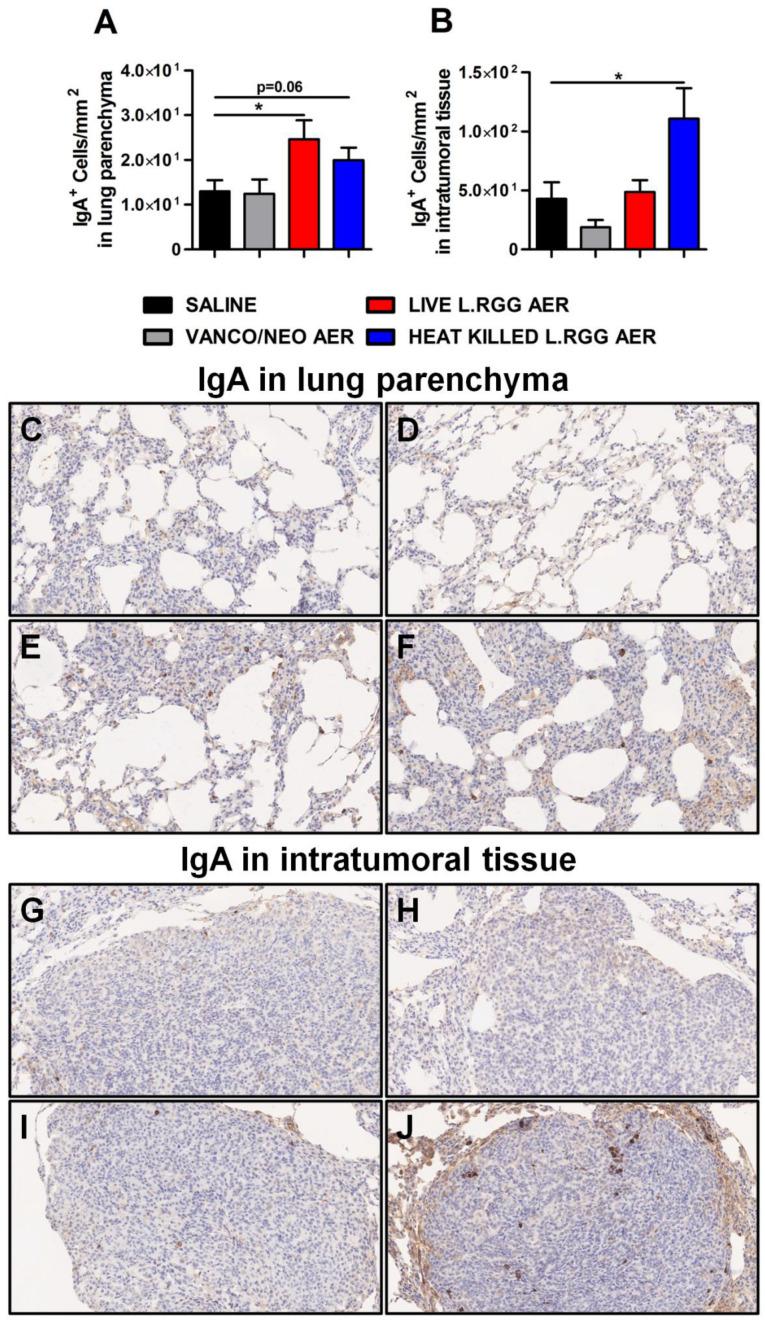
Impact of antibiotics and probiotic aerosolization on humoral immune response in the lung. Lung parenchyma (**A**) and intratumoral tissue (**B**) IgA protein level in formalin-fixed, paraffin-embedded lung specimens evaluated by IHC. Data are expressed as positive cells/mm^2^ (4 section/lung, 8–9 mice/group) (mean ± SEM). Lung parenchyma and intratumoral tissue representative staining with anti-human/mouse IgA antibody in saline- (**C**,**G**), vanco/neo- (**D**,**H**), live *L.RGG-* (**E**,**I**) and heat-killed *L.RGG-* (**F**,**J**) treated lungs are shown; 20× magnification. * *p* ≤ 0.05 by unpaired Student *t*-test.

Consistent with J chain expression and with the reported ability of nasal administration of *L.RGG* to stimulate mucosal humoral immunity [29,30,31,32], a higher presence of IgA^+^ cells was detected in lung parenchyma from *L.RGG*-treated mice (Figure 4A,E,F), even infiltrating the tumor nodules of heat-killed *L.RGG*-treated lung (Figure 4B,J).

**Figure 5:**
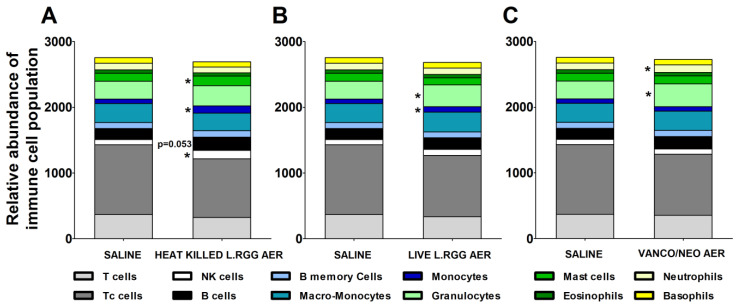
Immunological effects of vanco/neo and dead or live probiotic aerosolization on the lung microenvironment. Relative abundance of different immune cell populations in (**A**) heat killed *L.RGG*-, (**B**) live *L.RGG*- or (**C**) antibiotic-treated lungs obtained after TIMER 2.0 analysis of data from gene expression profile (9 samples/group) (mean ± SEM). * *p* ≤ 0.05 by unpaired Student *t*-test.

The effects of the different aerosol treatments on the tumor immune contexture were analyzed using TIMER 2.0 (http://timer.cistrome.org/) (accessed on 20 august 2022). The algorithm estimated a significant increase in B cells in heat-killed *L.RGG*-treated lungs, supporting the observed higher level of J chain and IgA proteins (Figure 5A). A significantly higher abundance of NK cells and mast cells was also observed in the same group (Figure 5A). Both aerosolized live and heat-killed *L.RGG* also significantly favored the infiltration of monocytes (Figure 5A,B). Moreover, the presence of granulocytes was significantly increased by the treatment with live *L.RGG* and vanco/neo that, among granulocytes, specifically determined an expansion of neutrophils (Figure 5B,C). No modulation in the other immune cell populations included in the TIMER 2.0 method was observed.

Overall, these results suggest that *L.RGG* aerosolization is able to affect the lung immune microenvironment, particularly stimulating humoral immunity.

### 2.3. L. rhamnosus GG-Up-Modulated J Chain Gene Is Associated with Survival of Lung Adenocarcinoma Patients

To investigate whether the *J chain* gene may have an influence on patient survival, *J chain* gene expression present in Kaplan–Meier plotter tool (https://kmplot.com/analysis/) (accessed on 10 october 2022) was utilized to divide cohorts of lung cancer patients (n = 1925) into two groups using the lower tertile as a cut-off, named low and high, and the association with patient survival was then calculated.

**Figure 6:**
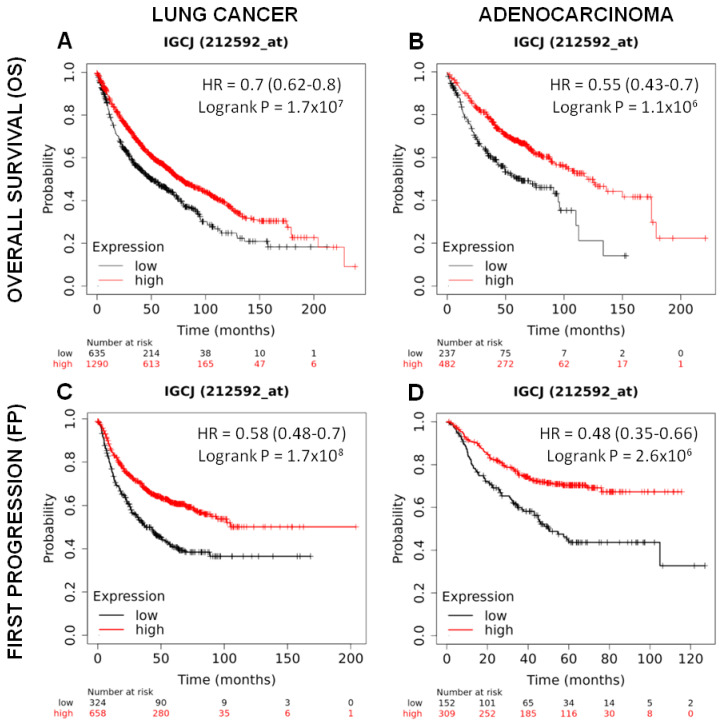
Prognostic significance of *J chain* expression on lung adenocarcinoma patients. Kaplan–Meier analysis of the prognostic value of *J chain* mRNA expression in the Kmplot (Affymetrix ID, 212592_at (Gene Symbol: IGCJ)). Overall survival curves plotted for patients with non-small cell lung cancer (n = 1925) (**A**) and with adenocarcinoma histotype (n = 719) (**B**). Time to first progression curves plotted for patients with non-small cell lung cancer (n = 982) (**C**) and with adenocarcinoma histotype (n = 461) (**D**). Cut-off value: lower tertile. HR, hazard ratio (with 95% confidence interval).

As shown in Figure 6A, lung cancer patients with high *J chain* gene expression showed significantly higher overall survival compared to those with low *J chain* expression (hazard ratio (HR): 0.7 95% confidence interval: 0.62–0.8 *p* = 1.7 × 10^7^). This association was maintained even restricting the analysis on adenocarcinomas (n = 719), the cancer subtype resembled by the murine tumor model induced by the urethane injection (HR: 0.55 95% confidence interval: 0.43–0.7 *p* = 1.1 × 10^6^) (Figure 6B).

A good prognosis according to high *J chain* expression was also observed considering the time to first progression in both lung cancer patients (n = 982) (HR: 0.58 95% confidence interval: 0.48–0.7 *p* = 1.7 × 10^8^) (Figure 6C) and adenocarcinoma patients (n = 461) (HR: 0.48 95% confidence interval: 0.35–0.66 *p* = 2.6 × 10^6^) (Figure 6D).

Multivariate analysis in a sub-cohort of lung cancer (n = 583, J chain univariate analysis HR = 0.38, 95% confidence interval: 0.29–0.51, *p* = 7.7 × 10^12^) (Table 3) and lung adenocarcinoma (n = 387, J chain univariate analysis HR = 0.41, 95% confidence interval: 0.27–0.61, *p* = 7.6 × 10^6^) (Table 4) patients in which smoking history and stage clinical variables, reported to be associated with prognosis [33,34], were available showed again a significant association between high *J chain* expression and better overall survival independently from all the other variables. Similar results were observed for the independent association between high *J chain* expression and increased time to first progression (Table 5 and Table 6) (n = 453 lung cancer patients, J chain univariate analysis HR = 0.46, 95% confidence interval: 0.33–0.63, *p* = 1.3 × 10^6^; and n = 384 lung adenocarcinomas patients, J chain univariate analysis HR = 0.47, 95% confidence interval: 0.33–0.67, *p* = 1.8 × 10^5^).

**Figure 7:**
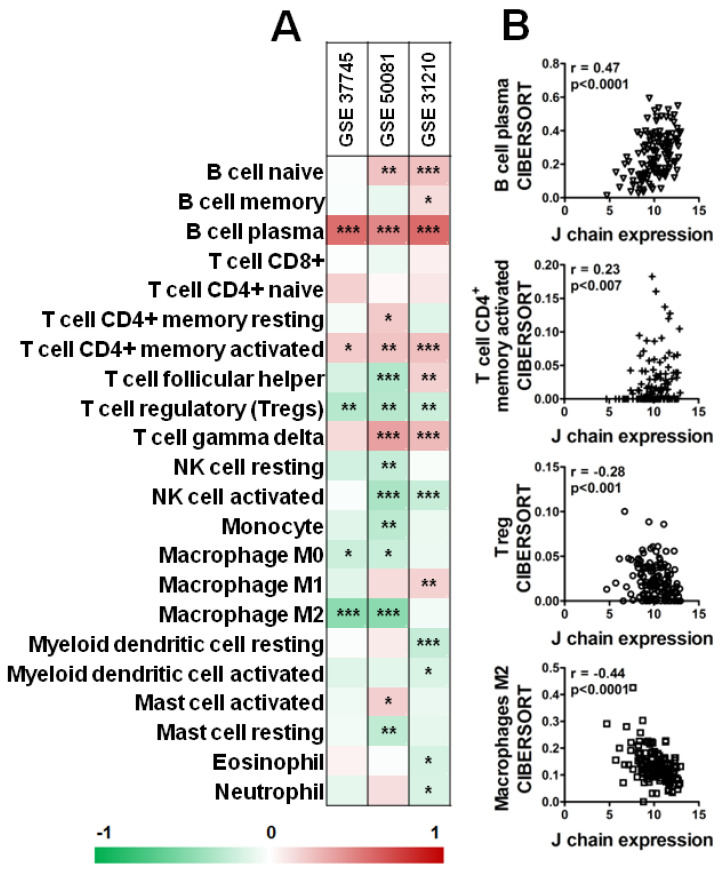
Correlation analysis of *J chain* expression and immune profile in lung adenocarcinoma patients. (**A**) Spearman correlation between *J chain* levels and relative fraction of 22 leukocyte subtypes (LM22 signature) as computed by CIBERSORT in adenocarcinoma samples of GSE37745 (n = 106), GSE50081 (n = 129), GSE31210 (n = 246) datasets. r of Spearman correlation is color-coded and relative significance is as follows: * *p* < 0.05, ** *p* < 0.01, *** *p* < 0.001. (**B**) Representative correlation plots in samples of the GSE50081 dataset between *J chain* expression and B plasma cells, CD4^+^ memory-activated T cells, Treg and M2 macrophages. r of Pearson and relative *p*-values are shown.

Interestingly, as shown in Figure 7, analysis of three datasets of KM plotter containing a higher number of samples (GSE_37745, GSE_50081, GSE_31210) by CIBERSORT revealed a significant positive correlation between B plasma cells and CD4+ memory-activated T cells and *J chain* expression level in all three datasets (Figure 7A,B). On the contrary, high *J chain* significantly correlated with reduced level of Tregs (Figure 7A,B). Of note, considering macrophage populations, we observed that high *J chain* level correlated with low immunosuppressive M2 macrophages (GSE_37745, GSE_50081) (Figure 7A,B) or with high antitumor M1 phenotype cells (GSE_31210) (Figure 7A).

Overall, these data reveal that the expression of J chain induced in mice by *L.RGG* aerosolization is associated with an increased overall survival and a favorable immune microenvironment in lung cancer patients.

## 3. Discussion

In the present work, we observed in a mouse model of urethane-induced lung carcinoma model that vancomycin/neomycin or probiotic *L.RGG* aerosol delivery weakened the tumor development induced by the exposure to the carcinogen. In the highly sensitive A/J mouse model, all the parameters used to measure tumor growth were strongly reduced by aerosol treatment with *L.RGG*. Vanco/neo aerosolization significantly impacted the diameter of macroscopic detectable nodules but did not affect their number, and only a slight reduction in the overall tumor area was observed. The evaluation of tumor grade also suggested a slower progression of tumors from adenoma to adenocarcinoma in all treated groups, as compared to control.

Similarly to that previously observed in mice aerosolized with vanco/neo [24], the antitumor effect induced by aerosol treatment with *L.RGG* might be mediated, at least partially, by a modulation of the lung microbiota, accordingly with the observed changes in microbiota richness and composition. Indeed, live *L.RGG* induced an over-representation of taxa known to stimulate an immune response by shaping the local immune microenvironment through the release of specific cytokines. For example, an increased abundance of *Corynebacteriaceae*, which have been described to stimulate the production of IL5, a potent activator of B cells and humoral responses [35], and of *Prevotellaceae*, reported to induce IL-23 and IL-1, have been observed [36]. Although further studies are needed to define the specific effects of heat-killed *L.RGG* on the lung microbiota, the microbial signals derived from dead microorganisms might also modulate the microbiome profile by interfering in the crosstalk between microbiota and immune cells through TLR-mediated activation of specific immune subsets able to shape the microbiota composition.

Accordingly, the antitumor effect of antibiotic and heat-killed *L.RGG* was paralleled by a reduction in immunosuppressive Tregs in lungs. This effect may be likely due to the pauperization or the imbalance induced by antibiotics or probiotics of microbial signals released from resident bacteria that influence Tregs recruitment and maintenance [24]. Accordingly, the interplay between microbiota-derived associated molecular patterns (MAMPs) ligands and airway-resident immune cells has been demonstrated to be an important factor that sustains immunosuppression. For instance, it has been reported that a previous exposure to a Toll-like receptor (TLR) ligand decreases the inflammatory responses to a second challenge with the same or a different ligand [37,38,39] and that chronic exposure to bacterial endotoxin promotes tolerogenic populations [40,41,42,43]. In the lung, specific strains of resident bacteria have been revealed to attenuate the immune response to lipopolysaccharide (LPS) [44], and the presence of commensal *Staphylococcus aureus* is essential for the resistance to acute inflammation induced by the influenza virus [45].

Since live *L.RGG* significantly reduced the number and dimension of tumor nodules and only induced a slight reduction in Tregs, our results indicate that other immune cell populations, activated by the aerosolized probiotic, contribute to *L.RGG* anti-cancer activity. In line with this view, we also observed that vanco/neo aerosolization, which also significantly reduced Tregs in the lung, had a lower antitumor effect than probiotic. Using the web source TIMER 2.0, we observed a modulation of B cells and various myeloid subsets, such as mast cells, monocytes and granulocytes, mostly in probiotic-treated lungs. These data are consistent with different studies demonstrating a modulation of mast cells [46], B cells [47], NK cells [48], monocytes [49] and granulocytes [50] by stimulation with probiotics. Moreover, more recently, supplementation of mice with *L.RGG* has been shown to induce CD8^+^ T cells through TLR2-mediated activation of DCs [51]; in melanoma and colorectal cancer murine models, *L.RGG* administration triggered type I interferon production in DCs enhancing the cross-priming of antitumor CD8^+^ T cells [52].

Therefore, it is possible to hypothesize that our probiotic aerosol treatments may induce a priming effect on the local immune response and a concomitant reduction in immunosuppressive cells. This hypothesis is corroborated by our previous published results [24] revealing a direct maturation of resident APCs in the lungs, the immune cells most responsive to microbial ligands through a wide expression of TLRs, and a reduction in pro-tumor M2 macrophages.

Our data revealed that the immunological effect exerted by *L.RGG* is higher compared to that of antibiotics. It also appeared that heat-killed *L.RGG* immunological activity is greater compared to that mediated by live bacteria. A possible explanation could be related to a wide and increased release of various MAMPs by dead bacteria that include ligands expressed on the cell surface but also molecules confined inside the bacterial cell that strongly activate specific TLRs. Although by definition, probiotics are live micro-organisms, a considerable number of studies demonstrated that the biological response obtained by live probiotics can be equally reached even with the administration of dead bacteria [53,54,55,56]. Different heat-killed strains of *Lactobacillus* have been shown to be able to stimulate the proliferation of murine splenocytes and the production of inflammatory cytokines [57,58,59,60,61]. Moreover, live and dead *Lactobacillus Rhamnosus GG* elicited an anti-inflammatory effect in arthritis and in inflammatory models in rats [60,62]. The major advantages for future applications of aerosolized dead bacterial cells in clinical settings are represented by the lack of proliferation capacity with a consequent increased safety compared to live probiotics, which could cause dangerous pathologies especially in oncologic immunodeficient patients, and the possibility to standardize the preparation of bacterial suspensions. Analysis of genes regulated by antibiotics or *L.RGG* aerosol showed the up-modulation of the *J chain* gene in both heat-killed- and live *L. Rhamnosus GG*-treated lungs compared to the other experimental groups. Consistent with these results, by IHC, we observed an increased level of J chain protein, even if not reflecting the difference in mRNA amounts observed between heat-killed and live *L. Rhamnosus GG*-treated lungs, and of IgA in lung sections and an increased estimated B cell infiltration by deconvolution analysis. J chain is the junction protein involved in the production of functionally active IgA and IgM [63], and many studies demonstrated the ability of different strains of bacteria, including *L.RGG*, to modulate the mucosal and humoral immunity in mice, promoting the development/maturation of B cells and immunoglobulin secretion [29,30,31,32]. For instance, *Enterococcus faecalis* and *Lactobacillus* spp. promote B-cell activation and stimulate IgA secretion in the intestine [64], while *Lactobacillus casei* Zhang [65] and *Lactobacillus crispatus KT-11* [66] induced an increase in IgA in intestinal fluid. In the lungs, pulmonary dendritic cells primed by TLR ligands of bacterial origin have been demonstrated to induce class-switch recombination in B cells and the generation of IgA-producing plasma cells [67]. It is not clear whether the increase in IgA and B cells induced by *L.RGG* in our study represents a response to bacteria exposure or to cancer cells; however, we speculate that, even in the case of a response to bacteria, modulation of the surrounding tumor microenvironment could make it permissive for antitumor effects and result in priming an antitumor immune response.

Interestingly, by in silico analysis on the KM plotter, we observed a significantly increased prognosis of lung adenocarcinoma patients with high *J chain* expression compared to those with low expression. Moreover, analyzing the three GEO datasets with the highest number of samples within the KM plotter, we observed a significantly positive correlation between *J chain* expression and the level of B plasma cells and CD4 T helper memory-activated cells, while an inverse correlation with immunosuppressive immune populations, such as Tregs and M2 macrophages, was detected. These results indicate that J chain expression is associated with a better survival that likely depends on a favorable activated immune microenvironment. An association between *J chain* expression and an increased OS has been observed in other tumor types, supporting our observation. Xin Feng et al. identified a gene signature that includes the *J chain* gene able to discriminate head and neck squamous cell carcinoma patients with better overall survival probability [68]. Moreover, recent studies revealed that a high J chain expression is associated with better prognosis in sarcoma [69] and breast cancer patients [70,71]. These data validate our hypothesis that the higher expression of J chain might reflect the activation of a local humoral immunity able to control tumor progression. Notably, our results in a murine model support that the presence of certain microbiota in patients influences the expression of J chain and the immune status. Hence, further studies are needed to investigate if J chain expression is associated with the presence of a specific microbiome profile in patients. These studies could pave the way for a possible clinical use of *L.RGG* or other probiotic(s) aerosolization to shape the microbial profile toward an immune-favorable context.

Overall, the results of this study highlight the efficacy of *L.RGG* aerosolization in reducing the growth of carcinogen-induced tumors in murine models by promoting the activation of the local immune microenvironment. Therefore, aerosol treatment with a probiotic could represent a feasible and non-invasive strategy to maintain a state of immunological alert and to counteract the establishment of an immune-suppressive lung microenvironment in patients at risk of development, such as smokers, individuals with low-risk nodules during monitoring and early-stage resected patients at risk of recurrence.

## 4. Materials and Methods

### 4.1. Mice and Experimental Protocols

A/J and BALB/c female mice were purchased from Jackson Laboratories (Bar Harbor, ME, USA) and Charles River Italia (Calco, Italy), respectively. Animals were maintained in laminar-flow rooms at constant temperature and humidity, with food and water given ad libitum. Mice were maintained under pathogen-free conditions at the animal facility of Fondazione IRCCS Istituto Nazionale dei Tumori.

Experiments were approved by the Ethics Committee for Animal Experimentation of the Fondazione IRCCS Istituto Nazionale dei Tumori of Milan according to institutional guidelines and to the Italian law (D-lgs 26/2014). In vivo experiments were approved by the Italian Ministry of Health.

At 4 weeks of age, animals were treated with a single intraperitoneal injection of urethane (1 g/kg of body weight) dissolved in water in order to induce the development of lung tumors [25,27]. Mice were treated with aerosol administration of vancomycin (PharmaTex, Milan, Italy) and neomycin (Sigma-Aldrich, Milan, Italy), or with the *L. Rhamnosus GG* probiotic (alive or heat-killed), starting 2 weeks after urethane injection for 14 weeks. For the *L. Rhamnosus GG* killing, bacteria cells were heated at 80 °C for 20 min and then stored in aliquots at −80 °C.

Aerosolization was performed using a tower inhalation system (IES 306 Inhalation Towers EMMS, Hampshire, UK) and the animals were treated 5 days/week with vanco/neo or 3 times/week with *L.RGG* (live or heat-killed). The control group was treated with saline. Vancomycin (50 mg) and neomycin (100 mg) were dissolved in 5 mL of saline, while *L.RGG* was resuspended in 10 mL of saline at the concentration of 10^9^ units/ml. The suspensions were placed in the nebulizer (Aeroneb Lab Micropump Nebulizer) (EMMS, Hampshire, UK) and used to treat groups of up to six mice by exposure to aerosol for 20 min.

The whole lung was excised and the number of lung nodules for each of five lobes per mouse was counted, and the diameter of each tumor was measured with a micrometer (graduation = 0.1 mm) under a stereomicroscope.

Lungs were then fixed in 10% neutral-buffered formalin for a minimum of 24 h and embedded in paraffin. Two 5 μm thick sections separated by 50 μm were cut from each lung lobe and stained with hematoxylin and eosin. The ratio between the tumor area and the area of the section for each section and each mouse was calculated and expressed as a %.

In addition, the number of pre-neoplastic and neoplastic lesions was assessed for each mouse, and each lesion was classified according to a grading scheme adapted from [72] into atypical adenomatous hyperplasia, adenoma, adenoma with areas of atypia and adenocarcinoma. In all experiments, mice were weighed and inspected for any sign of sufferance twice weekly and euthanized at 20 weeks, when their lungs were harvested.

### 4.2. Metagenomic Analysis of Bronchoalveolar Lavage

Bronchoalveolar lavage (BAL) was performed as described [73], in five euthanized mice/group aerosolized with saline or live *L.RGG* resuspended in 10 mL of saline at the concentration of 10^9^ units/mL for five days.

DNA extraction and sequencing of the 16S rRNA gene amplicons were performed by Vaiomer SAS (Labège, France) using a previously described optimized protocol [74]. The 16S rRNA gene profiling and taxonomic profiles were obtained as previously reported by Le Noci et al. [24].

### 4.3. Gene Expression Profiling and Immune Infiltrating Populations Analyses

RNA was extracted from two 50 μm lung tissue sections from FFPE tumor-bearing lung specimens using the FFPE RNeasy mini kit (QIAGEN, Milan, Italy). Gene expression profiling was performed by the Genomic Facility of Fondazione IRCCS Istituto Nazionale dei Tumori, Milan. After RNA extraction, quality check and quantification were performed by 4200 TapeStation (Agilent, Cernusco sul naviglio, Italy) and a Qubit fluorometer with the Qubit RNA HS assay kit (Thermo Fischer Scientific, Monza, Italy), respectively. RNA expression was assessed using the mouse Affymetrix Clariom S Pico assay (Thermo Fisher Scientific, Monza, Italy). A total of 100 ng of total RNA was used to generate the single-stranded cDNA samples for hybridization. Then, cDNA was enzymatically fragmented and biotinylated using the WT Terminal Labeling kit (Thermo Fisher Scientific, Monza, Italy), combined with hybridization buffer, and injected into Clariom S arrays targeting >20,000 well-annotated genes. The arrays were stained using the Affymetrix^®^ GeneChip^®^ Fluidics Station 450 and scanned with the 7G Affymetrix^®^ GeneChip^®^ Scanner 3000.

Raw data were processed using Transcriptome Analysis Console software (TAC v4.2.0) (Thermo Fisher Scientific, Monza, Italy). The Guanine Cytosine Count Normalization (GCCN) and Signal Space Transformation (SST) algorithms were applied to adjust the CEL file intensities. CEL files containing feature intensity values were converted into summarized expression values by robust multiarray average (RMA), which consists of background adjustment and quantile normalization across all chips. All samples passed quality control thresholds for hybridization, labeling and the expression of housekeeping gene controls.

The differential analyses of gene expression between treatment groups and saline controls, separately, were also performed in TAC software v4.0.2 by using default settings in terms of method, fold-change limits and significance level. Gene-specific differences in expression were considered statistically significant for false discovery rate (FDR)-corrected *p*-values ≤ 0.05.

The individual gene expression data obtained from TAC software processing were uploaded to the TIMER 2.0 web resource (http://timer.cistrome.org/) (accessed on 20 august 2022) to estimate the relative abundance of the immune cell infiltrate in the tumor-bearing lung from mice treated with antibiotics or probiotics by the MCP-counter algorithm.

The Kaplan–Meier plotter (https://kmplot.com/analysis/index.php?p=service&cancer=lung) ((accessed on 10 October 2022) [75] was used for univariate and multivariate analysis to assess the association of J chain mRNA expression with overall survival (OS) and first progression (FP) in lung cancer patients and lung adenocarcinoma subtype using the lower tertile as a cut-off to split patients into low and high *J chain* expression groups. Multivariate analysis was performed considering tumor stage and the smoking status as covariates.

To define the immune landscape of lung adenocarcinoma according to *J chain* expression, microarray processed data of the GSE_31210, GSE_50081 and GSE_37745 public datasets were retrieved from the GEO repository (http://www.ncbi.nlm.nih.gov/gds/) (accessed on 14 September 2022). Multiple probes mapped to the same gene were collapsed in each dataset, selecting the probe with the highest interquartile range. Then, datasets were uploaded to the TIMER 2.0 web resource (http://timer.cistrome.org/) (accessed on 11 October 2022) applying the LM22 signature of the CIBERSORTx tool. Relative fractions of each of the 22 immune populations were correlated with *J chain* expression levels in adenocarcinoma samples by Spearman correlation analysis.

### 4.4. Immunohistochemistry Analysis

Immunohistochemistry was performed on four non-consecutive 5 μm sections from FFPE tumor-bearing lungs specimens to assess the immune cell infiltration. Sections underwent deparaffinization and heat-induced epitope retrieval for 40 min at 96 °C (Dewax and HIER Buffer H, Thermo Scientific, Runcorn, UK, cat. no. TA-999-DHBH). Endogenous peroxidase activity was blocked by incubating sections in 3% H2O2 for 10 min. Slides were rinsed, incubated with phosphate-buffered saline (PBS) containing 10% normal serum for 30 min at room temperature to reduce nonspecific background staining, and then incubated for 1 h at room temperature with specific primary antibodies. Sections were then incubated with a biotinylated secondary antibody, labeled by the avidin–biotin–peroxidase procedure (VECTASTAIN Elite ABC-Peroxidase Kit Standard, Vector Laboratories, cat. no. VC-PK-6100-KI01). The immunoreaction was visualized with 3,30-diaminobenzidine (DAB, Peroxidase DAB Substrate Kit, Vector Laboratories, cat. no. VC-SK-4100-KI01) substrate, and sections were counterstained with Mayer’s hematoxylin. Known positive control sections were included in each immunolabeling assay. Details of primary antibodies and reagents used for the immunohistochemical procedure are reported in the following Table 7.

IHC-stained slides were separately evaluated in tumoral and parenchymal areas, using QuPath v0.3.2 image analysis software [76]. For tumoral areas, each lesion was manually annotated. For parenchymal areas, 4 microscopic fields at 20× were randomly selected for evaluation. The number of positive cells for each marker was assessed in the selected regions using the “positive cell detection” algorithm, and the results were expressed as the number of positive cells per mm^2^ of area.

### 4.5. Statistical Analysis

Differences among groups from all in vivo and in vitro experiments were compared using a two-tailed unpaired Student’s t-test and considered significant at *p* ≤ 0.05. All analyses were performed using GraphPad Prism version 5.0 for Windows (GraphPad Software).

## Figures and Tables

**Table 1:** Effect of dead *L.RGG* aerosol treatment on transcriptomic profile of the lung. Genes differentially expressed in comprehensive gene expression profiles comparing RNA extracted from heat-killed *L.RGG*-treated lungs *versus* saline-treated lungs. Gene expression average per group was calculated using one-step Tukey’s bi-weight algorithm, reported as binary logarithm (log2). The fold-change can be calculated as two power of the absolute difference between group averages. A negative fold-change indicates a greater expression in the control group. Further details on the calculations are available on pages 11–12 of the Affymetrix Statistical Algorithms Description Document, http://tools.thermofisher.com/content/sfs/brochures/sadd_whitepaper.pdf (accessed on 15 June 2022) ((FDR *p*-value < 0.05) (8/9 samples/group). Abbreviations: Avg, average; SD, standard deviation; FDR, false discovery rate.

Gene Symbol	Description	Heat killed L.RGG Aer, Avg (SD), log2	Saline, Avg (SD), log2	Fold Change	P-val	FDR P-val
Dnajc11	DnaJ (Hsp40) homolog, subfamily C, member 11	8.59 (0.74)	9.97 (0.29)	−2.60	1.01 × 10^6^	0.0112
Jchain	immunoglobulin joining chain	10.69 (1.61)	7.66 (0.82)	8.21	8.15 × 10^6^	0.0303
Vmn1r73	vomeronasal 1 receptor 73	6.31 (0.72)	5.19 (0.23)	2.18	3.11 × 10^5^	0.0406
Six1	sine oculis-related homeobox 1	11.89 (0.89)	10.26 (0.95)	3.10	3.48 × 10^5^	0.0419
Gm3468	predicted gene 3468	9.66 (1.08)	8.50 (0.35)	2.24	4.86 × 10^5^	0.0461
Atp6v0d2	ATPase, H+ transporting, lysosomal V0 subunit D2	8.69 (0.52)	10.11 (0.69)	−2.67	5.21 × 10^5^	0.0461
Scimp	SLP adaptor and CSK interacting membrane protein	7.34 (0.45)	8.42 (0.60)	−2.11	7.84 × 10^5^	0.0461
Gm16506	predicted gene 16506	7.65 (0.72)	6.63 (0.26)	2.03	9.12 × 10^5^	0.0461
Zdhhc21	zinc finger, DHHC domain containing 21	8.24 (0.88)	9.32 (0.35)	−2.11	2.00 × 10^4^	0.0485
Rhox8	reproductive homeobox 8	6.33 (0.82)	5.32 (0.33)	2.01	2.00 × 10^4^	0.0485
Git1	G protein-coupled receptor kinase-interactor 1	7.29 (0.64)	8.41 (0.42)	−2.19	2.00 × 10^4^	0.0485

**Table 2:** Effect of live or dead *L.RGG* aerosol treatment on transcriptomic profile of the lung. Genes differentially expressed in comprehensive gene expression profiles comparing RNA extracted from live *L.RGG*-treated lungs *versus* saline-treated lungs. Gene expression average per group was calculated using one-step Tukey’s bi-weight algorithm, reported as binary logarithm (log2). The fold-change can be calculated as two power of the absolute difference between group averages. A negative fold-change indicates a greater expression in the control group. Further details on the calculations are available on pages 11–12 of the Affymetrix Statistical Algorithms Description Document, http://tools.thermofisher.com/content/sfs/brochures/sadd_whitepaper.pdf (accessed on 15 June 2022) (FDR *p*-value < 0.05) (8/9 samples/group). Abbreviations: Avg, average; SD, standard deviation; FDR, false discovery rate.

Gene Symbol	Description	Live L.RGG Aer, Avg (SD), log2	Saline, Avg (SD), log2	Fold Change	P-val	FDR P-val
Jchain	immunoglobulin joining chain	11.87 (1.17)	7.66 (0.82)	18.49	2.20 × 10^8^	0.0005

**Table 3:** Multivariate analysis considering overall survival in lung cancer patients. Multivariate analysis of *J chain* gene expression and smoking history and stage on overall survival in lung cancer (n = 583) patients.

Multivariate Analysis in Lung Cancer Patients (OS)		
	*p* Value	Hazard Ratio
Stage	0	1.66 (1.35–2.03)
Smoking history	0.055	0.65 (0.41–1.01)
Jchain gene	0	0.51 (0.38–0.7)

**Table 4:** Multivariate analysis considering overall survival in lung adenocarcinoma patients. Multivariate analysis of *J chain* gene expression and smoking history and stage on overall survival in lung adenocarcinoma (n = 387) patients.

Multivariate Analysis in Lung Adenocarcinoma Patients (OS)		
	*p* Value	Hazard Ratio
Stage	0	2.42 (1.77–3.31)
Smoking history	0.0308	0.59 (0.37–0.95)
Jchain gene	0.0011	0.5 (0.33–0.76)

**Table 5:** Multivariate analysis considering first progression in lung cancer patients. Multivariate analysis of *J chain* gene expression and smoking history and stage on time to first progression in lung cancer (n = 453) patients.

Multivariate Analysis in Lung Cancer Patients (FP)		
	*p* Value	Hazard Ratio
Stage	0	2.12 (1.63–2.76)
Smoking history	0.8614	1.03 (0.71–1.51)
Jchain gene	0.0008	0.56 (0.4–0.79)

**Table 6:** Multivariate analysis considering first progression in adenocarcinoma patients. Multivariate analysis of *J chain* gene expression and smoking history and stage on time to first progression in lung adenocarcinoma (n = 384) patients.

Multivariate Analysis in Lung Adenocarcinoma Patients (FP)		
	*p* Value	Hazard Ratio
Stage	0	2.16 (1.61–2.89)
Smoking history	0.7501	0.94 (0.64–1.39)
Jchain gene	0.0017	0.55 (0.38–0.8)

**Table 7:** List of primary antibodies used in the immunoistochemisty analysis.

Primary Antibody	Clone	Supplier and Code	Clonality	Working Dilution	Secondary Antibody	Supplier and Code	Working Dilution
FoxP3	FJK-16S	eBioscience (14-5773-82)	Rat monoclonal	1:200	Anti-rat IgG	Vector (VC-BA-4001-MC05)	1:200
IgA alpha chain	\	Prodotti Gianni Srl (ab97233)	Goat polyclonal	1:1500	Anti-goat IgG	Vector (VC-BA-5000-MM15)	1:200
J chain	SP105	Invitrogen (MA5-16419)	Rabbit monoclonal	1:500	Anti-rabbit IgG	Vector (VC-BA-1000-MM15)	1:200

## Data Availability

The raw sequencing data of gene expression profiling are available on the Gene Expression Omnibus with the code GSE215317.

## Supplementary Materials

The following supporting information can be downloaded at: https://www.mdpi.com/article/10.3390/ijms232112748/s1.
