# Supplementary material for: Live or Heat-Killed Lactobacillus rhamnosus Aerosolization Decreases Adenomatous Lung Cancer Development in a Mouse Carcinogen-Induced Tumor Model
Source: Int J Mol Sci. 2022 Oct 22;23(21):12748. doi: 10.3390/ijms232112748 (PMC9656640; doi:10.3390/ijms232112748)
Supplement: Supplementary file 1 [file ijms-23-12748-s001.zip › ijms-1954276-supplementary.pdf]

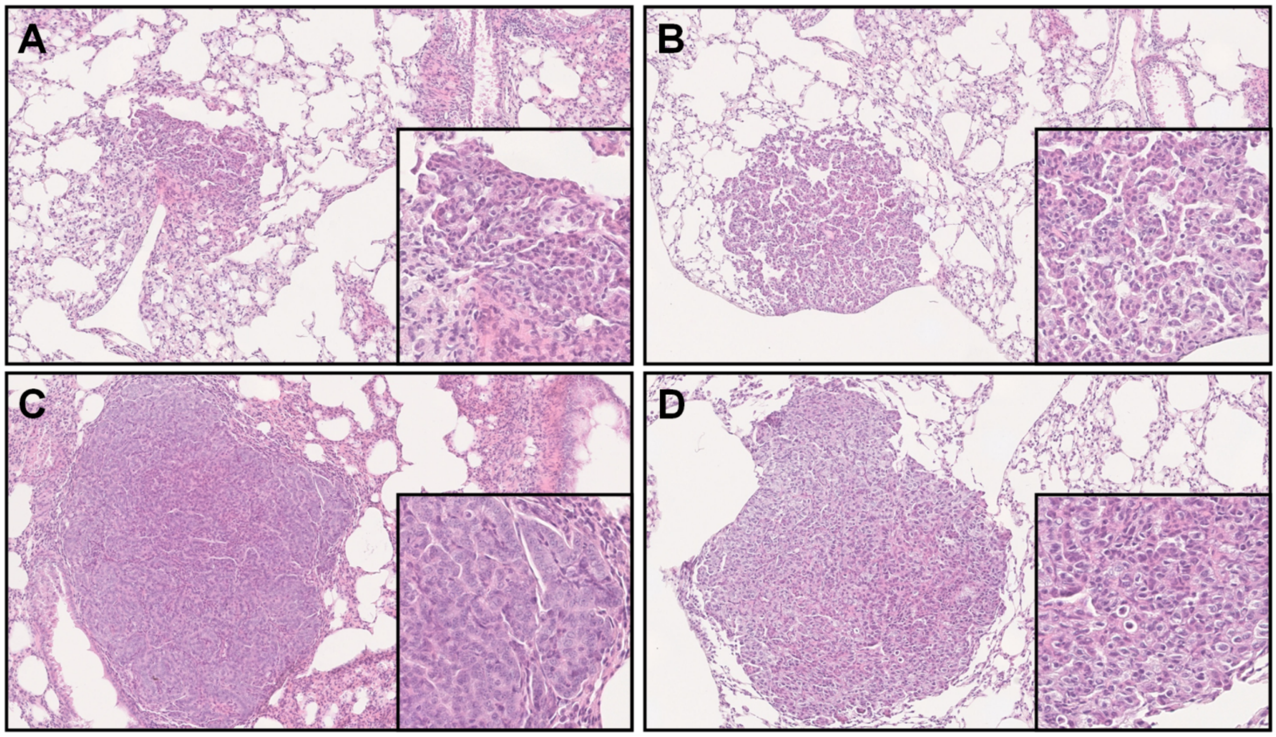

Figure S1. Histopathological evaluation of urethane-induced lung lesions.
Representative examples of lung lesions induced by urethane treatment. **(A)** Atypical adenomatous hyperplasia, **(B)** adenoma. **(C)** adenoma with areas of atypia and **(D)** well differentiated adenocarcinoma. Hematoxylin and Eosin, 10x. Inset 40x.

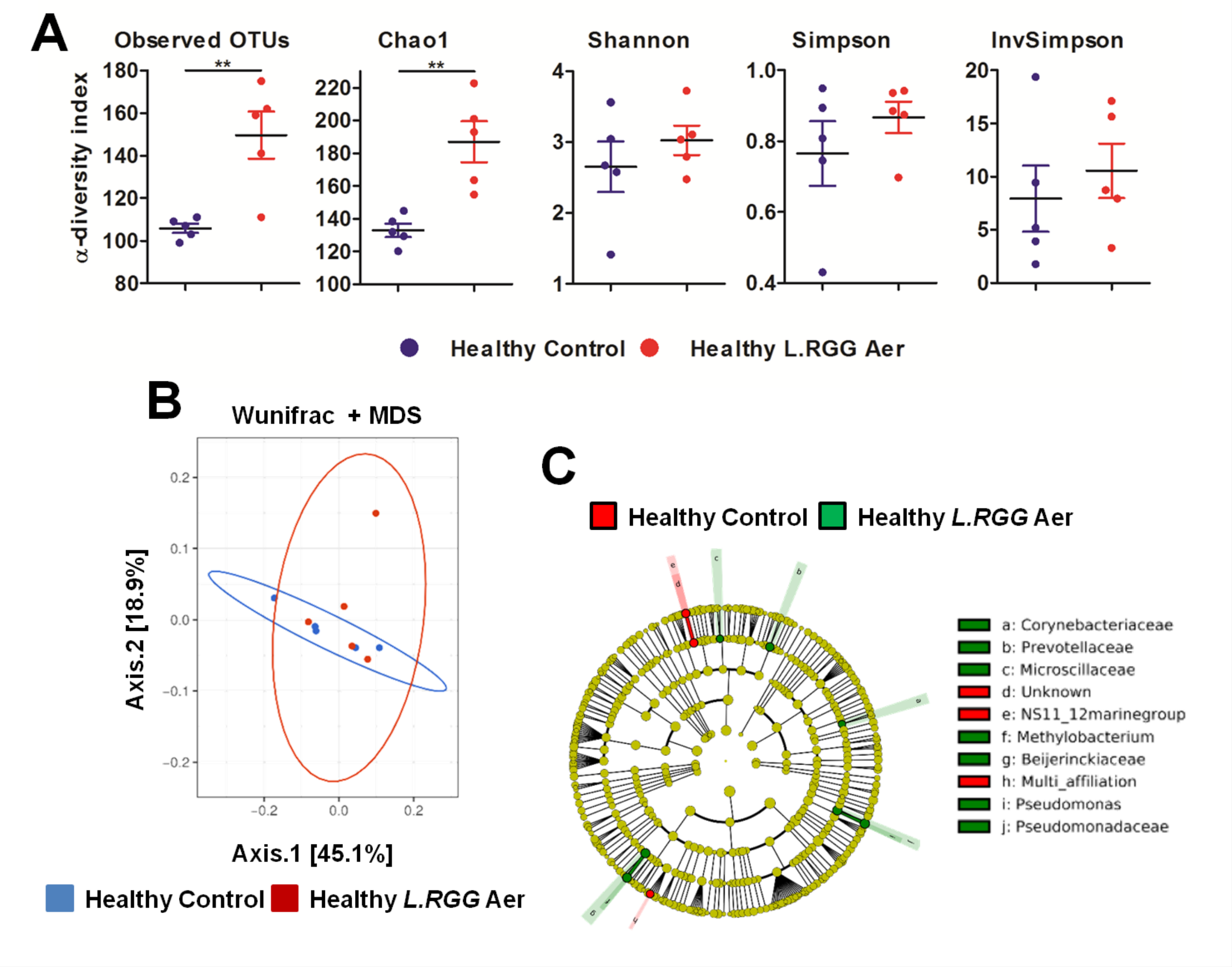

Figure S2. Metagenomic analysis of healthy lung after *L.RGG* aerosol treatement.
16S rRNA gene profiling was used to compare the bacterial community in healthy lung samples from mice not treated or treated with live *L.RGG* aerosolization. **(A)** Intra-subject bacterial richness and evenness (α-diversity) analyzed using observed OTUs, Chao1, Shannon, Simpson, and inverse Simpson algorithms (5 samples/group) (mean ± SEM). **(B)** Inter-sample (β-) diversity analysis carried out with the WUniFrac algorithms plus multidimensional scaling (MDS) (5 samples/group). **(C)** Cladogram representation derived from linear discriminant analysis score computed for differentially abundant taxa in the lung bacteria of untreated compared with live *L.RGG* treated healthy mice. Regions in red indicate taxa enriched in healthy control mice, whereas regions in green indicate taxa enriched in healthy *L.RGG* treated mice. $^{**}p < 0.01$ by unpaired student t-test.

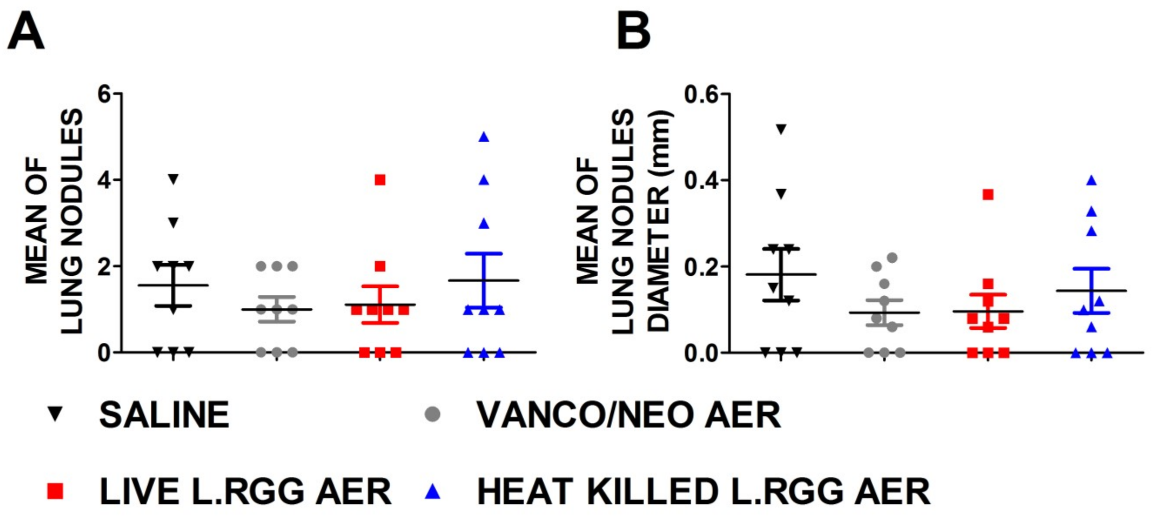

Figure S3. Impact of aerosol treatment with vancomycin/neomicin or *L.RGG* on adenocarcinoma development induced by urethane in low sensitive BALB/c mice model.
Mean number **(A)** and mean diameter **(B)** of lung tumor nodules in BALB/c mice i.p. injected with urethane and aerosolized with saline, vanco/neo, live *L.RGG* and killed *L.RGG* (9 mice/group) (mean ± SEM).
